# Exosomes Derived from miR-146a-5p-Enriched Mesenchymal Stem Cells Protect the Cardiomyocytes and Myocardial Tissues in the Polymicrobial Sepsis through Regulating MYBL1

**DOI:** 10.1155/2021/1530445

**Published:** 2021-10-15

**Authors:** Chun Liu, Jianhua Xue, Bo Xu, Aixian Zhang, Lili Qin, Jiajia Liu, Yang Yang

**Affiliations:** ^1^Department of Emergency Medicine, Affiliated Hospital of Nantong University, No. 20 Xisi Road, Chongchuan District, Nantong City, Jiangsu Province 226001, China; ^2^Department of Trauma Center, Affiliated Hospital of Nantong University, Nantongy, Jiangsu Province 226001, China; ^3^Department of Orthopaedics, Qidong Hospital of Traditional Chinese Medicine, Nantong City, Jiangsu Province 226200, China; ^4^Department of General Practice Medicine, Affiliated Hospital of Nantong University, Nantong City, Jiangsu Province 226001, China; ^5^Department of Endoscopic Center, Affiliated Hospital of Nantong University, Nantong, Jiangsu Province 226001, China

## Abstract

**Background:**

At present, the study has confirmed that the mesenchymal stem cell-derived exosomes (MCSs-Exo) possess cardio-protection in sepsis. Nevertheless, the molecular mechanism of the protection of MSCs-Exo in sepsis remains unknown. Therefore, this research is aimed at studying the molecular mechanism.

**Methods:**

The effects of MSCs-Exo and miR-146a-5p in LPS-induced cardiomyocytes (H9C2 cells) *in vitro* were verified by CCK-8, EdU assay, flow cytometry, Western blot assay, and RT-qPCR. The effect of MSCs-Exo *in vivo* was evaluated by CLP-induced sepsis model. The potential gene in MSCs-Exo was verified by bioinformatics analysis, and the potential target of miR-146a-5p was identified by bioinformatics analysis and luciferase reporter assay. At last, the function of miR-146a-5p and its target genes on LPS-induced cardiomyocytes (H9C2 cells) *in vitro* was validated by recuse experiment.

**Results:**

Our findings revealed that MSCs-Exo could effectively protect cardiomyocytes of inflammation model *in vitro* and myocardial tissues of sepsis model *in vivo*. Meanwhile, we found that miR-146a-5p was a potential gene in MSCs-Exo, and MYBL1 was the target gene of miR-146a-5p and negatively regulated by miR-146a-5p. In addition, miR-146a-5p overexpression promoted proliferation and inhibited apoptosis of LPS-induced cardiomyocytes. The rescue experiment demonstrated that miR-146a-5p could effectively repress the inflammatory response of cardiomyocytes via decreasing MYBL1 expression.

**Conclusion:**

This study suggests that miR-146a-5p-bearing MSC-derived exosomes may become an effective treatment for sepsis.

## 1. Introduction

Sepsis is an uncontrolled inflammatory response induced by pathogens, which leads to severe local infection and even multiple organ dysfunction syndromes, ultimately death [1]. It is reported that 20 million patients are diagnosed with sepsis every year worldwide, and 30% of them lose their lives [2]. At present, accumulated studies have shown that 40% of patients with sepsis has observed the cardiac injury and dysfunction, which indicates that cardiac injury and dysfunction are fatal complications of sepsis [3, 4]. Therefore, exploring the new molecular mechanism or new therapies to resolve the complex clinical syndrome caused by sepsis is a hot research topic.

Mesenchymal stem cells (MSCs) were originated from bone marrow or adipose tissue which could differentiate into different cells including osteoblasts, chondrocytes, adipocytes, and myoblasts under the appropriate microenvironment condition [5]. Several studies have demonstrated that MSCs have multiple biological functions. For example, owing to MSC differentiation capability into various cell lineages, it was able to promote the injured tissue regeneration. The molecular mechanism of MSCs on tissue regeneration was MSC paracrine signaling which was mediated by proteins such as cytokines and chemokines with anti-inflammatory, antioxidative, antiapoptotic, and proangiogenic properties [6–8]. In addition, MSCs also could regulate immune response, promote angiogenesis, inhibit fibrosis, and protect the nervous system [9]. Recently, numerous studies have demonstrated that MSCs effectively improve myocardial function and reduce mortality in the animal models of polymicrobial sepsis induced by cecal ligation and puncture (CLP) [10–14]. Firstly, MSCs interacted with host macrophages to reduce the secretion of proinflammatory cytokines (TNF-*α*, IL-1*β*, IL-6, and TGF-*β*) in order to relieve cardiac injury and dysfunction [13]. Then, MSCs could differentiate into endothelial cells, vascular smooth muscle cells, and cardiac-like myocytes to repair and regenerate the cardiac tissues [14]. These evidences indicated that MSCs had a protective effect on cardiomyocytes and tissues. Yet, the function of MSC-mediated cardio-protection in sepsis is unclear.

Recently, for one thing, as the paracrine effectors of MSCs, exosomes have gradually become the research hot. Exosomes are nanosized vesicles containing various macromolecular substances with the size of approximately 30-150 nm that mediate local or systemic intercellular communication [15, 16]. Besides, exosomes can transport proteins and a specific set of functional RNAs to target cells or tissues to perform long distance cell signal transduction process [17]. For another, as the paracrine effectors of MSCs, exosomes derived from MSCs have various biological functions in various diseases. In cancer field, exosomes acted as a bridge during the interaction between MSCs and tumor cells. MSC-derived exosomes (MSCs-Exo) promoted renal cancer progression through modulating the tumor microenvironment, and MSC-derived exosomes facilitated nasopharyngeal carcinoma occurrence and development via activating the FGF19/FGFR4 signaling pathway and modulating the epithelial-mesenchymal transition (EMT) [18, 19]. In the cardiovascular disease field, the bone barrow MSC-derived exosomes inhibited the myocardial infarction pathogenesis through autophagy [20]. MSC-derived exosomes also decreased the myocardial ischemia and reperfusion injury [21]. Besides, MSC-derived exosomes was regarded as an effective method to treat the ischemic disease [22, 23]. Nonetheless, the function of MSC-derived exosomes in sepsis-induced cardiac dysfunction is still obscure.

In general, the biological function of exosomes depends on the exosomal composition (miRNAs, mRNAs, and proteins). Among them, microRNAs (miRNAs) are the most important exosomal components and play a key role in the regulation of target cells or tissues by exosomes [24]. MiRNAs, short noncoding RNAs containing about 22~25 nucleotides, could regulate downstream genes expression at a posttranscriptional level [25]. So far, several studies have researched the miRNA functionality in MSC-derived exosomes: miR-181a in MSCs-Exo affected the inflammatory response when myocardial ischemia-reperfusion was injured [26]; exosomes derived from miR-29b-modified bone narrow mesenchymal stem cells (BMSCs) could repair spinal cord injury (SCI) in rat model [27]; miR-204 repressed osteogenesis and promoted adipogenesis of mesenchymal progenitor cells and BMSCs through regulating Runx2 [28]. Meanwhile, miR-146a-5p in MSCs-Exo exerts important role in cardiovascular disease. For instance, miR-146a-5p in hUMSCs-Exo reduced microglial-mediated neuroinflammatory response via inhibiting the IRKA1/TRAF6 signaling pathway in ischemic stroke [29]. MiR-146a-5p in BMSC-derived exosome could alleviate the intracerebral hemorrhage (ICH) through repressing neuronal apoptosis and microglial M1 polarization [30]. However, the cardioprotection mechanism of exosomes derived from miR-146a-5p-enriched MSCs in sepsis-induced cardiac dysfunction remains unknown.

In the study, we firstly explored the function of MSC-derived exosomes on cardiomyocytes *in vitro* and *in vivo*. The results showed that MSC-derived exosomes could effectively protect cardiomyocytes in LPS-induced cardiomyocyte inflammation *in vitro* and contribute to cardio-protection in CLP-induced mice sepsis model *in vivo*. Through the bioinformatics analysis, we found that MSC-derived exosomes exerted the cardio-protection in sepsis through regulating miR-146a-5p and miR-146a-5p further protected the cardiomyocyte through regulating MYBL1. These findings will provide a novel therapeutic approach to treating the sepsis.

## 2. Materials and Methods

### 2.1. Animals

All the C57BL/6 mice were purchased from the Laboratory Animal Center of Nanjing University (Nanjing, China). The animal experiments were approved by the Ethics Committee of Affiliated Hospital of Nantong University. The mice were housed under specific pathogen-free condition, bred with a 12 h light/dark cycle and had free access to food and water.

### 2.2. Cell Culture and Transfection

The rat cardiomyocyte H9C2 and rat bone marrow-derived mesenchymal stem cells (BMMSCs) were purchased from American Type Culture Collection (ATCC). The H9C2 cells and BMMSCs cells were cultured in DMEM-H (Keygen, Nanjing, China) medium and DMEM (Keygen, Nanjing, China) medium, respectively. All the cells were incubated in the humified atmosphere at 37°C with 5% CO_2_. The H9C2 cells were transfected with 2 *μ*g pcDNA-MYLB1, 2 *μ*g pcDNA-NC, 50 nM miR-146a-5p mimics, and 50 nM NC mimics (all purchased from Shanghai GenePharma Co., Ltd.) for 48 h at 37°C using Lipofectamine 2000 (Thermofisher, USA).

### 2.3. Isolation and Characterizations of MSC-Derived Exosomes

The Total Exosomes Isolation Reagent (Invitrogen, CA) was used to isolate exosomes from BMMSCs. After 48 h of culture in exosome-free medium, the BMMSCs were centrifuged at 3000 rpm and 4°C for 25 min to ensure removal of cell debris and dead cells, followed by filtration through a 0.2 mm filter. The resulting solution was centrifuged at 12000 rpm and 4°C for 2.5 h and centrifuged again at 12000 rpm and 4°C for 2 h. The collected exosomes were resuspended in PBS and extracted for subsequent use. To determine the characteristics of exosomes, the respective levels of the specific surface markers heat shock protein 70 (Hsp70, ab45133), CD63 (ab59479), and CD81 (ab155760) were measured by Western blot assay. Exosome size was determined by Zetasizer Nano ZS (Malvern Instruments, Malvern, UK). The exosomes were then allowed to settle on copper grids coated with Forvar and carbon. Copper grids were immersed in 2% phosphotungstic acid for 1 min. The morphology of exosomes was analyzed under a transmission electron microscope (TEM, Tecnai Spirit; FEI, Hillsboro Oregon, USA).

### 2.4. CCK-8 Assay

For cell viability, 1 × 10^6^ cells were seeded into a 6-well plate for 0, 24, 48, and 72 h, and then, 10 *μ*L CCK-8 reagent (Beyotime, Shanghai, China) was added. The cell viability was detected by measuring the absorbance at 450 nm using a microplate reader (BioTek Instruments Inc., Winooski, VT, USA).

### 2.5. EdU Incorporation Assay

For cell proliferation, 1 × 10^6^ cells per well were plated into 24-well plates. After transfection, the cells were incubated with EdU (100 *μ*L) for 2 h, fixed with 4% paraformaldehyde for 30 minutes. The nuclei were counterstained by DAPI solution (Keygen, Nanjing, China). Proliferation cells were monitored under fluorescent microscope (Leica, Wetzlar, Germany).

### 2.6. Flow Cytometry Assay

Cell apoptosis was measured by flow cytometry assay. Briefly, the transfected H9C2 cells (1 × 10^6^ cells) in each treatment group were collected and washed with PBS. Each precipitate was resuspended in 400 *μ*L PBS. After that, the Annexin V-FITC (5 *μ*L, Keygen, Nanjing, China) and PI staining solution (5 *μ*L, Keygen, Nanjing, China) were added and incubated in dark for 30 min. At last, the cell apoptotic rate was examined by flow cytometry (FACSCalibur, BD Biosciences) and was analyzed by FlowJo software (Version 7.6.1).

### 2.7. Reverse Transcription-Quantitative PCR (RT-qPCR)

Total RNAs were extracted from H9C2 cells, MSC-derived exosomes, or exosomes-treated samples and purified by using the TRIzol reagent (ThermoFisher, USA). Reverse transcription was performed in accordance with the instruction of the one-step miRNA reverse transcription kits (Takara, Tokyo, Japan) and complementary DNA (cDNA) reverse transcription kits (Takara, Tokyo, Japan). RT-qPCR assay was performed with SYBR Green PCR Master Mix (Takara). The primer sets for each gene are as follows: miR-146a-5p, forward 5′-GGCCTGAGAACTGAATTCCATGG-3′, reverse 5′-GTGCAGGGTCCGAGGTATTC-3′; MYBL1, forward 5′-GTCAGCTGAGAATGAAGTTAGA-3′, reverse 5′-AGAAGAATCAGGCACTCAACTG-3′; GADPH, forward 5′-GAGAAGGCTGGGGCTCATTT-3′, reverse 5′-AGTGATGGCATGGACTGTGG-3′; U6, forward 5′-CGCTTCGGCAGCACATATAC-3′, reverse 5′-TTCACGAATTTGCGTGCAT-3′.

The fold change in gene expression was calculated by 2^−ΔΔCT^ method, and GADPH and U6 were used as internal controls.

### 2.8. Western Blot Assay

All the total proteins were extracted by RIPA kit (Beyotime, Shanghai, China), and the protein concentration was analyzed by BCA assay kit (Beyotime, Shanghai, China).

The proteins were separated by SDS-PAGE (Keygen, Nanjing, China) and transferred onto 0.22 *μ*m PVDF (Beyotime, Shanghai, China). The membranes were incubated with primary antibodies (Abcam, Cambridge, MA, UK). After 4°C incubation overnight, the membranes were incubated with HRP-conjugated secondary antibodies (Sigma, Aldrich, 1 : 2000) for 1 h at 37°C. The protein bands were visualized by using the Image Lab (Bio-Rad Laboratories, CA, USA).

Dilution ratio of the antibodies used in this experiment was as follows: primary antibodies: anti-CD63 (ab59479), 1 : 1000; anti-CD81 (ab155760), 1 : 1000; anti-Hsp70 (ab45133), 1 : 1000; anti-cleaved caspase-3 (ab214430), 1 : 2000; anti-cleaved caspase-9 (ab2324), 1 : 2000; anti-MYBL1 (ab23458), 1 : 2000; anti-*β*-actin (ab8227), 1 : 2000.

### 2.9. Measurement of Lactate Dehydrogenase (LDH) and Creatine Kinase (CK) in Peripheral Blood and Cell Culture Supernatants

The activities of LDH and CK were measured using the LDH detection kit and CK detection kit (Nanjing Jiancheng Biological Product, Nanjing, China), respectively. Briefly, after the cells was treated for 24 h, the supernatant was harvested for the measurement of LDH and CK levels. After CLP surgery for 24 h, the blood samples were collected through using heparinized needles in cardiac puncture, and then, the blood samples were centrifuged at 8000 rpm for 10 min to collect serum. The serum was employed to detect the LDH and CK levels. Absorbance was detected at 450 nm and 660 nm, respectively, using a microplate reader (Molecular Devices, Sunnyvale, CA, USA).

### 2.10. MSC-Derived Exosomes Labeling

The MSCs-Exo were labelled with the fluorescent dye PKH26 (Umibio, China) according to manufacturer's instructions. Then, the stained exosomes were coincubated with LPS-induced H9C2 cells. After 24 h incubation, the nuclei of each group of cells were stained with DAPI. Finally, the laser scanning confocal microscope (Nikon, Japan) was used to observe and record the red fluorescence of PKH26 in each group of cells.

### 2.11. Cecal Ligation and Puncture (CLP) Polymicrobial Sepsis Model

CLP was used to induce polymicrobial sepsis in mice as previously described [31]. Briefly, the mice were anesthetized employing isoflurane and underwent laparotomy. A midline incision was made on the anterior abdomen, and the cecum was exposed and ligated with a suture. Two punctures were made through the cecum with a 28-gague needle, and feces were extruded from the holes. Then, the abdomen was closed. After surgery, fluid resuscitation was administered subcutaneously in mice.

All the mice were divided into three groups: (1) control group: the mice that were not subjected to surgery; (2) CLP group; (3) CLP + MSCs-Exo group: the CLP-mice were treated with MSCs-Exo.

### 2.12. Histological Analysis

After the mice were executed, the myocardial tissues were dissected, washed, and fixed with 4% paraformaldehyde at room temperature for 48 h before being embedded in paraffin. The 4 *μ*m sections were prepared, stained with hematoxylin and eosin, and viewed by a light microscope (BX53, Olympus Corporation).

### 2.13. Dual-Luciferase Reporter Gene Assay

The sequences of MYBL1-wild type and MYBL1-mutated type were inserted into the pmiRGLO reporter vector (Synthgene Biotech, Nanjing, China). Subsequently, cells were cotransfected with miR-146a-5p mimics or mimic-NC together with WT or Mut. After 48 h of the transfections, the luciferase activity was measured by using the dual-luciferase reporter assay (Promega, Shanghai, China). Renilla luciferase activity was used as an internal control.

### 2.14. Statistical Assay

SPSS 19.0 (IBM Crop., Armonk, NY, USA) was used to data analysis. Three independent experiments were repeated, and the data are presented as mean ± the standard deviation (SD). Specifically, Student's *t*-test or one-way ANOVA test followed by Tukey's post hoc test was executed to compare the differences between two groups or more. *P* < 0.05 was considered to indicate a statistically significant difference.

## 3. Results

### 3.1. Isolation and Identification of MSC-Derived Exosomes (MSCs-Exo)

**Figure 1:**
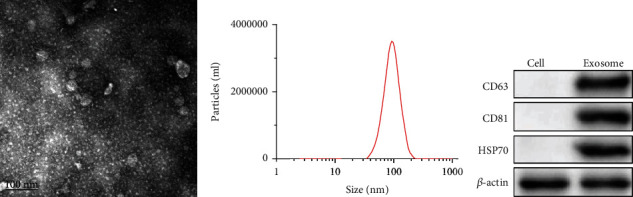
Isolation and identification of MSC-derived exosomes (MSCs-Exo). (a) The morphology of MSCs-Exo was detected by transmission electron microscope (TEM). (b) The concentration particles of MSCs-Exo were detected by nanoparticle tracking analysis (NTA). (c) The surface marker protein levels of CD63, CD81, and Hsp70 of MSCs-Exo were detected by Western blot assay.

The MSCs-Exo were isolated from BMMSCs. Then, we characterized the MSC-derived exosomes. Firstly, we employed TEM and NTA to character the morphology and concentration particles of MSC-derived exosomes. The results of TEM analysis indicated that the isolated exosomes had a spherical vesicle-like structure with an average diameter between 30 and 150 nm (Figure 1(a)). The outcomes of NTA revealed that the average particles size of exosomes was 100 nm (Figure 1(b)). In addition, we detected the surface marker proteins CD63, CD81, and Hsp70 of exosomes through Western blot assay. The results revealed that the surfaces of exosomes highly expressed CD63, CD81, and Hsp70 (Figure 1(c)). These evidences confirmed that MSC-derived exosomes were successfully isolated.

### 3.2. Effects of MSCs-Exo on the Proliferation and Apoptosis of Cardiomyocytes *In Vitro*

**Figure 2:**
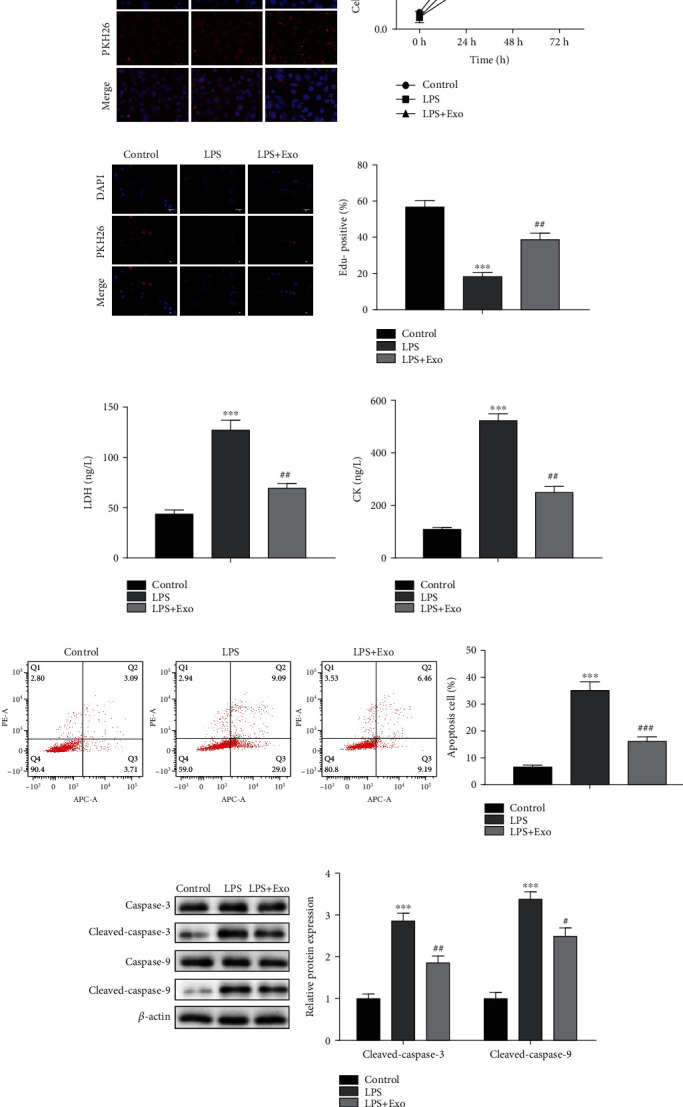
Effects of MSCs-Exo on the proliferation and apoptosis of cardiomyocytes *in vitro*. (a) Red dye PKH26-labeled MSCs-Exo was detected in LPS-induced cardiomyocytes H9C2 cells. (b) The cell viability was detected by CCK-8 assay. (c) The cell proliferation was examined by EdU assay. (d) The release levels of LDH and CK were measured by LDH detection kit and CK detection kit, respectively. (e) The cell apoptosis was assessed by flow cytometry assay. (f) The protein levels of caspase-3, cleaved-caspase-3, caspase-9, and cleaved-caspase-9 were detected by Western blot assay. ^∗∗∗^*P* < 0.001, ^#^*P* < 0.05, ^##^*P* < 0.01, ^###^*P* < 0.001 vs. the control group.

In order to explore whether MSCs-Exo was absorbed by cardiomyocytes H9C2 *in vitro*, we labeled MSCs-Exo with the red fluorescent membrane dye PKH26 and incubated the PKH26-MSCs-Exo with LPS-induced H9C2 cells. Meanwhile, the PKH26 was incubated with H9C2 cells and LPS-induced H9C2 cells, respectively. After incubated for 24 h, we used the laser confocal microscope to evaluate the red fluorescence intensity of PKH26 in three different cell groups (control group, LPS group and LPS + Exo group). The results indicated that the red fluorescence in LPS + Exo group was the strongest which suggested that LPS-induced H9C2 cells had the capacity of specific uptake the MSCs-Exo (Figure 2(a)). Then, we intended to study whether the MSCs-Exo protected the function of cardiomyocytes after being taken up by LPS-induced H9C2 cells. To this end, we studied the effect of MSCs-Exo on cardiomyocytes proliferation through CCK-8 and EdU assay. After the 72 h coincubation with MSCs-Exo, the cell viability of LPS + Exo group was markedly increased compared to the LPS group. However, the cell viability of LPS + Exo group and LPS group was lower than the control group (Figure 2(b)). The results of EdU staining were the same as above. The proportion of EdU positive cells in the LPS+ Exo group was higher than LPS group (Figure 2(c)). Lactate dehydrogenase (LDH) and creatine kinase (CK) are enzymes originally existed in the cytoplasm. When the cardiomyocytes were injured, LDH and CK could enter the tissue fluid or blood stream from the cardiomyocytes. Therefore, LDH and CK could be regarded as the biomarkers of myocardial injury. In this study, we detected the LDH and CK levels in cell culture supernatant. The results indicated that LDH and CK levels were obviously increased in the LPS group and LPS + Exo group while treated with MSCs-Exo markedly decreased LDH and CK levels compared to the LPS group, which suggested that MSCs-Exo relieved LPS-induced cardiomyocytes injury (Figure 2(d)). After that, we studied the effect of MSCs-Exo on cardiomyocytes apoptosis. The results indicated that the apoptosis ratio in LPS and LPS + Exo group and the cleaved-caspase-3 and cleaved-caspase-9 expression in LPS and LPS + Exo group were markedly increased as compared with the control group. Nevertheless, the LPS + Exo group could effectively reduce the apoptosis ration and cleaved-caspase-3 and cleaved-caspase-9 proteins expression when compared with LPS group (Figures 2(e) and 2(f)). These evidences demonstrated that MSCs-Exo could protect cardiomyocytes when myocardium was damaged.

### 3.3. Effects of MSCs-Exo on CLP-Induced Mice Sepsis Model *In Vivo*

**Figure 3:**
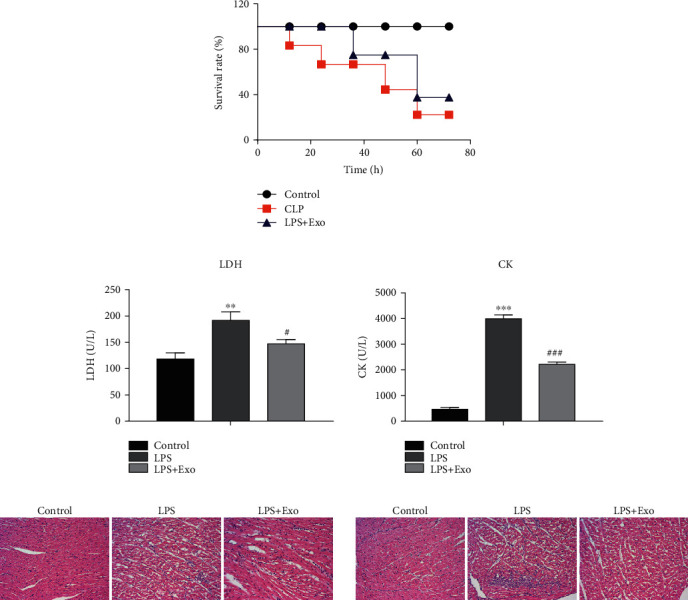
Effects of MSCs-Exo on CLP-induced mice sepsis model *in vivo*. (a) The survival rate of CLP-mice was significantly improved via MSCs-Exo treatment. (b) The release levels of LDH and CK in CLP-mice and CLP-mice treated with MSCs-Exo were measured by LDH detection kit and CK detection kit, respectively. (c) Hematoxylin and eosin staining in CLP-treated mice. ^∗∗^*P* < 0.01, ^∗∗∗^*P* < 0.001, ^#^*P* < 0.05, ^###^*P* < 0.001 vs. the control group.

In order to explore the effects of MSCs-Exo cardiac-protection in sepsis, we constructed the polymicrobial sepsis model using applied cecal ligation and puncture method in mice. When the sepsis model was constructed, we firstly evaluated the animal survival after treatment of CLP mice with MSCs-Exo. The results indicated that the CLP mice treated with MSCs-Exo obviously improved the mice survival rate as compared to the CLP mice group (Figure 3(a)). In addition, we detected the release levels of LDH and CK in mice serum. The LDH and CK levels were markedly improved in CLP mice group and CLP + MSCs-Exo mice group while treated with MSCs-Exo in CLP mice greatly reduced the serum levels of LDH and CK when compared with the CLP mice group (Figure 3(b)). At last, to study the effect of MSCs-Exo on myocardial architecture, the cardiomyocyte crosssectional areas via hematoxylin and eosin staining were detected. The results indicated the CLP group disrupted myocardial fibers and increased cardiomyocyte crosssectional areas while treated with MSCs-Exo could effectively reduce the cardiomyocyte crosssectional areas (Figure 3(c)). Based on the above results, MSCs-Exo could validly protect cardiac cells and cardiac tissues in mice with sepsis.

### 3.4. MSCs-Exo Repressed the Progression of Sepsis through miR-146a-5p

**Figure 4:**
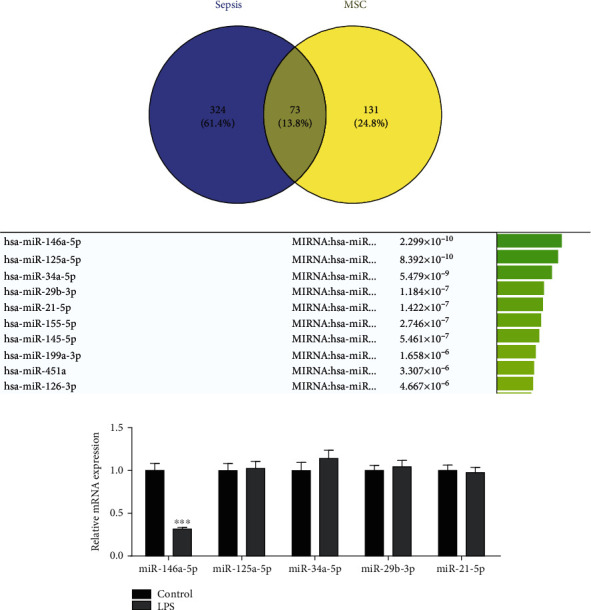
MiR-146a-5p is the potential target of MSCs-Exo in sepsis. (a) The comparative toxicogenomics database and GeneCards database were used to identify the potential target of MSCs-Exo. (b) The profiler cluster analysis was employed to select gene with higher expression from the selected target genes with transcriptional activity. (c) The expression levels of the top five miRNAs (miR-146a-5p, miR-125a-5p, miR-34a-5p, miR-29b-3p, and miR-21-5p) in sepsis model were detected by RT-qPCR. ^∗∗∗^*P* < 0.001 vs. the control group.

To verify the regulatory mechanism of MSCs-Exo in sepsis, we employed the bioinformatics analysis. From the comparative toxicogenomics database (http://ctdbase.org/), 324 targets related to sepsis were obtained. 131 targets of MSCs-Exo were acquired by GeneCards database (http://www.genecards.org/). Crosswise, a total of 73 related targets of MSCs-Exo in sepsis were identified (Figure 4(a)). To further screen out effective targets from the 73 identified targets, the profiler cluster analysis was employed. The results indicated that 10 miRNAs were the main target genes of MSCs-Exo in sepsis (Figure 4(b)). Then, we detected the expression levels of the top five miRNAs (miR-146a-5p, miR-125a-5p, miR-34a-5p, miR-29b-3p, and miR-21-5p) in sepsis model. Among them, miR-146a-5p expression was lower in CLP group as compared to control group while other four miRNAs expression level in CLP group were consistent with control group (Figure 4(c)).

**Figure 5:**
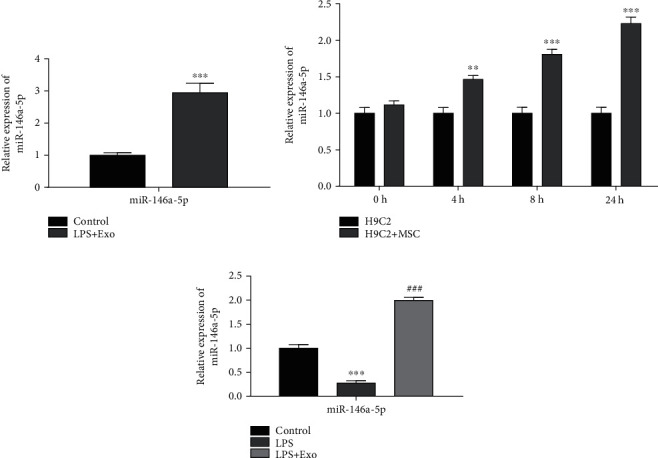
MSCs-Exo repressed the progression of sepsis through miR-146a-5p. (a) The expression level of miR-146a-5p in MSCs-Exo was measured by RT-qPCR. ^∗∗∗^*P* < 0.001 vs. the control group. (b) The expression level of miR-146a-5p in cardiomyocyte H9C2 cells and cardiomyocyte H9C2 cells treated with MSCs-Exo was detected by RT-qPCR. ^∗∗^*P* < 0.01, ^∗∗∗^*P* < 0.001 vs. the H9C2 cells. (c) The expression level of miR-146a-5p in CLP-induced sepsis model and CLP-induced sepsis model treated with MSCs-Exo was examined by RT-qPCR. ^∗∗∗^*P* < 0.001, ^###^*P* < 0.001 vs. the control group.

In order to further hypothesize whether MSCs-Exo inhibited sepsis via miR-146a-5p, we firstly detected miR-146a-5p expression in MSCs-Exo by RT-qPCR. The results indicated that miR-146a-5p expression was increased in MSCs-Exo (Figure 5(a)). Meanwhile, after treatment with MSCs-Exo in cardiomyocyte H9C2 cells and CLP-induced sepsis model in mice, we detected the miR-146a-5p expression in cardiomyocyte and myocardial tissues, respectively. The miR-146a-5p expression was obviously increased in cardiomyocyte and myocardial tissues (Figures 5(b) and 5(c)). These above conclusions indicated that MSCs-Exo repressed the progression of sepsis through miR-146a-5p *in vitro* and *in vivo.*

### 3.5. Effects of miR-146a-5p Overexpression on LPS-Induced Sepsis Model *In Vitro*

**Figure 6:**
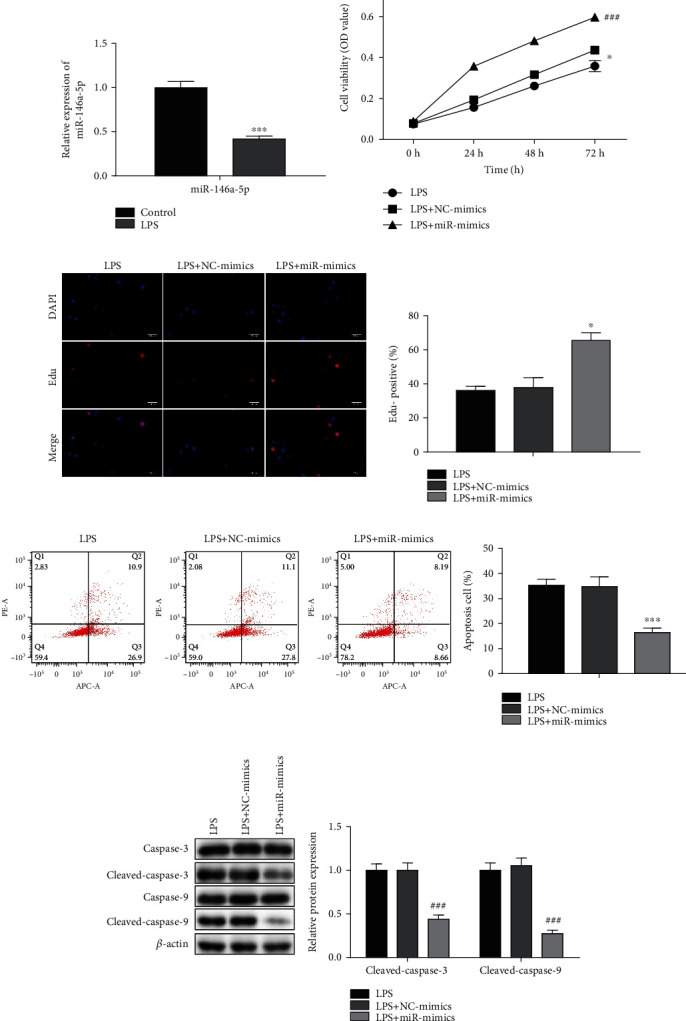
Effects of miR-146a-5p overexpression on LPS-induced sepsis model *in vitro*. The NC mimics and miR-146a-5p mimics were transfected into the LPS-induced H9C2 cells. (a) The expression level of miR-146a-5p in LPS-induced H9C2 cells was examined by RT-qPCR. ^∗∗∗^*P* < 0.001 vs. the control group. (b) The cell viability was detected by CCK-8 assay. (c) The cell proliferation was measured by EdU assay. (d) The cell apoptosis was assessed by flow cytometry assay. (e) The protein levels of caspase-3, cleaved-caspase-3, caspase-9, and cleaved-caspase-9 were detected by Western blot assay. ^∗^*P* < 0.05, ^∗∗∗^*P* < 0.001, ^#^*P* < 0.05, ^###^*P* < 0.001 vs. the LPS group.

To explore the role of miR-146a-5p in LPS-induced sepsis model *in vitro*, we firstly examined the miR-146a-5p expression in LPS-induced H9C2 cells. The miR-146a-5p expression was reduced in LPS-induced H9C2 cells. Therefore, the NC mimics and miR-146a-5p mimics were transfected into LPS-induced H9C2 cells, respectively. From the CCK-8 and EdU assay, compared to the control group, miR-146a-5p overexpression significantly improved cell viability and proliferation (Figures 6(b) and 6(c)). Besides, the results of flow cytometry and Western blot analysis indicated that the miR-146a-5p overexpression could effectively decrease the H9C2 cell apoptosis ratio and the cleaved-caspase-3 and cleaved-caspase-9 expression as compared to the control group (Figures 6(d) and 6(e)). These evidences indicated that miR-146a-5p overexpression effectively protected cardiomyocytes when myocardium was inflamed.

### 3.6. MYBL1 Was the Target of miR-146a-5p

**Figure 7:**
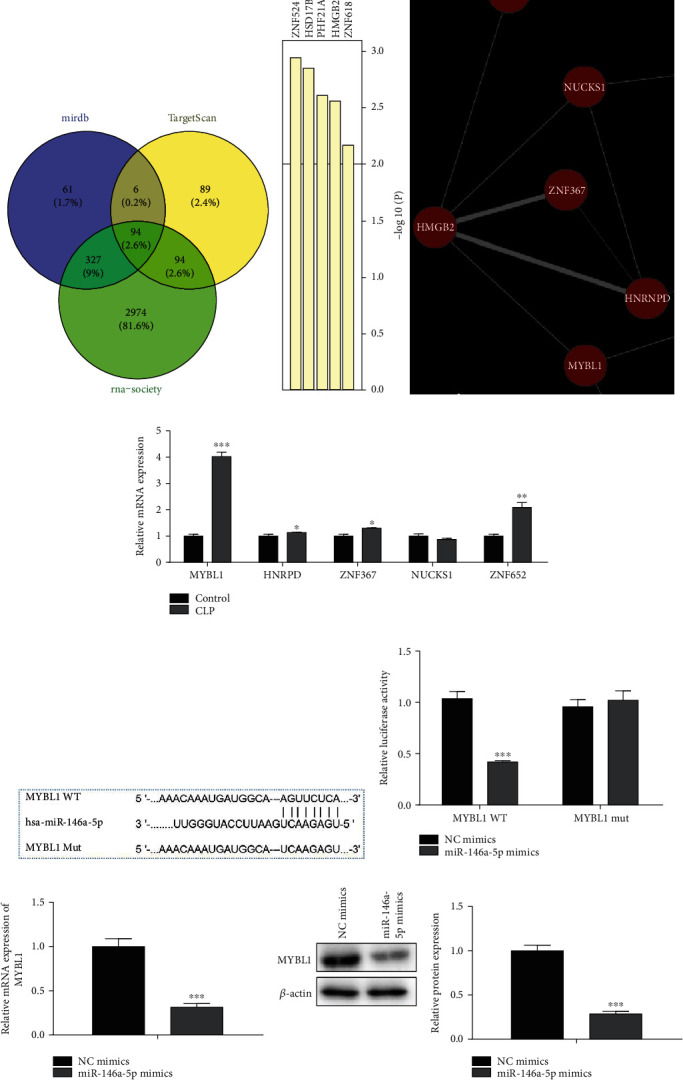
MYBL1 was the target of miR-146a-5p. (a) The Mirdb, Targetscan, and RNA-Society databases were employed to identify the target of miR-146a-5p. (b) Five potential target (MYBL1, HNRPD, ZNF637, NUCKS1, and ZNF652) of miR-146a-5p were screened by profiler cluster analysis. (c) The expression levels of MYBL1, HNRPD, ZNF637, NUCKS1, and ZNF652 in sepsis model were detected by RT-qPCR. ^∗^*P* < 0.05, ^∗∗^*P* < 0.01, ^∗∗∗^*P* < 0.001 vs. the control group. (d) The predicted miR-146a-5p binding sites in the region of MYBL1 and the corresponding mutant sequence were shown. (e) The relative values of luciferase signal. (f) The relative expression of MYBL1 in LPS-induced H9C2 cells after transfected with NC mimics and miR-146-5p mimics, respectively. (g) The protein expression of MYBL1 in LPS-induced H9C2 cells after transfected with NC mimics and miR-146-5p mimics, respectively. ^∗∗∗^*P* < 0.001 vs. the NC mimics.

To study the potential molecular mechanism of miR-146a-5p, we predicted the differentially targets which were related with the progression of sepsis by bioinformatics analysis (Mirdb, Targetscan and RNA-Society). Through this assay, five potential targets (MYBL1, HNRPD, ZNF637, NUCKS1, and ZNF652) were screened (Figures 7(a) and 7(b)). Then, we detected the five potential target expressions in LPS-induced H9C2 cells. Among them, MYBL1 was highly expressed which suggested that MYBL1 was a target of miR-146a-5p (Figure 7(c)). Meanwhile, the 3′-UTR of the MYBL1 contained a putative binding site of miR-146a-5p (Figure 7(d)). From the luciferase reporter assay, miR-146a-5p overexpression decreased the luciferase activity of MYBL1-WT while the luciferase activity of MYBL1-Mut had no distinct changes (Figure 7(e)). In addition, miR-146a-5p overexpression repressed the MYBL1 expression at both transcriptional and translational levels (Figures 7(f) and 7(g)). Therefore, MYBL1 was a target gene of the miR-146a-5p and was negatively regulated by miR-146a-5p.

### 3.7. MiR-146a-5p Inhibited the Sepsis Induced by LPS Treatment through Regulating MYBL1 *In Vitro*

**Figure 8:**
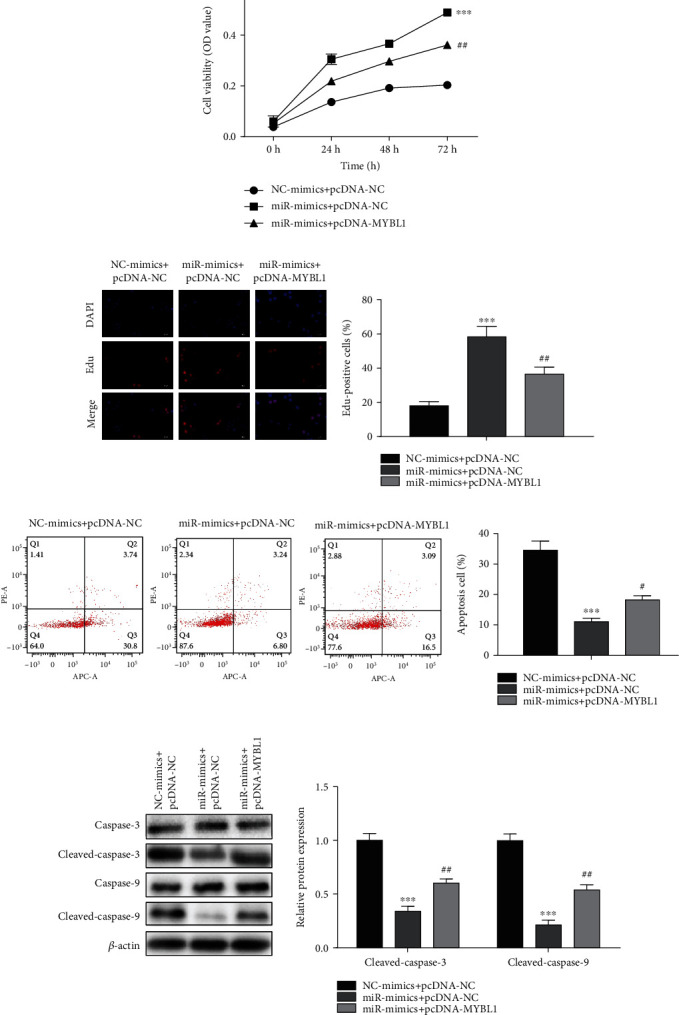
MiR-146a-5p inhibited the sepsis induced by LPS treatment through regulating MYBL1 *in vitro.* The pcDNA-MYBL1 and pcDNA-NC were transfected into LPS-induced H9C2 cells treated with miR-146a-5p mimics. (a) The cell viability was detected by CCK-8 assay. (b) The cell proliferation was examined by EdU assay. (c) The cell apoptosis was assessed by flow cytometry assay. (d) The protein levels of caspase-3, cleaved-caspase-3, caspase-9, and cleaved-caspase-9 were detected by Western blot assay. ^∗∗∗^*P* < 0.001, ^#^*P* < 0.05, ^##^*P* < 0.01 vs. the NC mimics + pcDNA-NC.

To further verify if MYBL1 mediating the inhibition of sepsis caused by miR-146a-5p, the pcDNA-MYBL1 and pcDNA-NC were transfected into LPS-induced H9C2 cells treated with miR-146a-5p mimics. The CCK-8 and EdU assays showed that the pcDNA-MYBL1 suppressed the increased of cell viability and proliferation caused by miR-146a-5p mimics (Figures 8(a) and 8(b)). Flow cytometry and Western blot assay revealed that the apoptosis ratio and the protein levels of cleaved-caspase-3 and cleaved-caspase-9 of LPS-induced H9C2 cells were reduced by miR-146a-5p mimics, but this alteration was reversed by MYBL1 overexpression (Figures 8(c) and 8(d)). Therefore, upregulation of MYBL1 could promote the progression of sepsis inhibited by miR-146a-5p mimics in vitro.

## 4. Discussion

Recently, some researches have revealed that the sepsis-induced cardiac injury and dysfunction are related to the high mortality [3, 4]. Myocardial circulation disorders, direct myocardial depression, and mitochondrial dysfunction are the main pathophysiological features of sepsis-induced cardiac dysfunction. Although the septic cardiomyopathy is a research hotspot all the time, the clinical treatment effect of sepsis-induced cardiac dysfunction is disappointing, and the prognosis is poor. The main reason is that the study of molecular mechanism and therapeutic methods of septic cardiomyopathy is still incomplete [32, 33]. In recent years, some evidences have indicated that apoptosis is associated with the pathogenesis of cardiac dysfunction caused by sepsis [34–36]. Besides, some substances exerted protective effects in sepsis-induced cardiac dysfunction, such as resveratrol [37], melatonin [38], and exosomes [39]. Exosomes, a nanosized vesicle, can mediate cell signal process by transporting proteins and a specific set of functional RNAs to target cells or tissues [15–17]. As the paracrine effectors of MSCs, MSCs-Exo have exerted important roles in cardiovascular disease [20–23]. For instance, MSCs-Exo promoted cardiac repair through modulating miR-125b in myocardial infarction [40]. In this study, we evaluated the effects of MSCs-Exo on cardiomyocytes *in vitro* and *in vivo*. In LPS-induced cardiomyocyte inflammation model *in vitro*, MSCs-Exo promoted the proliferation and inhibited apoptosis of cardiomyocyte. In CLP-induced mice sepsis model *in vivo*, MSCs-Exo could effectively protect myocardium. Moreover, cytokines originally existed in the cytoplasm. When the myocardium was injured caused by sepsis, cytokines were leaked from cardiomyocyte into the tissue fluid or blood stream. Therefore, cytokines were identified as the main mediators of myocardial depression in sepsis due to their release [41]. Based on this situation, we detected the LDH and CK levels in cell culture supernatant and serum. The LDH and CK level were reduced after treated with MSCs-Exo. These findings showed that MSCs-Exo could validly protect cardiac cells and cardiac tissues in sepsis-induced cardiac dysfunction.

Currently, at the genetic level, the biological function of MSCs-Exo depends on the regulation of mRNA and miRNA [24]. MiR-124a-overexpressing MSCs-Exo could repress the proliferation and migration of rheumatoid arthritis-related fibroblast-like synoviocyte cells and MiR-150-5p-overexpressing MSCs-Exo inhibited synoviocyte hyperplasia and angiogenesis to reduce joint destruction [42, 43]. MiR-146a-5p, belongs to the member of miR-146a family, has multiple biological functions including anticancer, anti-inflammatory, and antioxidative [44, 45]. In cardiovascular disease, miR-146a-5p in MSC-derived exosomes could reduce the neuroinflammatory response in ischemic stroke and alleviate the intracerebral hemorrhage (ICH) [29, 30]. In this study, through bioinformatics analysis, we found that miR-146a-5p served as the main gene to regulate the inhibitory effect of MSCs-Exo on sepsis. Meanwhile, the miR-146a-5p expression was reduced in the sepsis model and upregulated in the MSCs-Exo. Besides, the miR-146a-5p expression was markedly increased in cardiomyocyte and myocardial tissues after treatment with MSCs-EXO in cardiomyocyte H9C2 cells and CLP-induced sepsis model. What is important, miR-146a-5p overexpression could protect cardiomyocytes when myocardium was injured. In conclusion, MSCs-Exo could inhibit the progression of sepsis through miR-146a-5p *in vitro* and *in vivo*, and miR-146a-5p overexpression repressed the inflammatory response *in vitro*.

MYB protooncogene-like 1(MYBL1), located on chromosome 8q13.1, is a novel tumor-related gene and a strong activator of transcription [46]. Multiple researches revealed that MYBL1 contributed to the occurrence and development of cancer, such as hepatocellular carcinoma, glioma, and adenoid cystic carcinoma [47–49]. For example, MYBL1 promoted the hepatocellular carcinoma growth and metastasis via upregulation of TWIST1 [47]. However, the role of MYBL1 in sepsis-induced cardiac dysfunction and inflammatory response is rarely reported. In this study, through bioinformatics analysis, luciferase reporter assay, and RT-qPCR, we found that MYBL1 was a target of the miR-146a-5p and negatively regulated by miR-146a-5p. Meanwhile, the rescue experiment revealed that upregulation of MYBL1 promoted the progression of LPS-induced inflammatory response caused by miR-146a-5p overexpression *in vitro*.

In conclusion, we demonstrated that MSCs-Exo could effectively protect cardiomyocytes of inflammation model *in vitro* and myocardial tissues of sepsis model *in vivo*. Meanwhile, we confirmed that miR-146a-5p served as the main gene to regulate the inhibitory effect of MSCs-Exo on sepsis. More importantly, we verified that MYBL1 was the target of miR-146a-5p, and miR-146a-5p inhibited the progression of inflammatory response via modulating MYBL1 *in vitro*. Therefore, this research indicates that miR-146a-5p-bearing MSC-derived exosomes may become an effectively method to treat the sepsis.

## Data Availability

All data generated or analysed during this study are included in this published article.
